# Correlations between forest soil quality and aboveground vegetation characteristics in Hunan Province, China

**DOI:** 10.3389/fpls.2022.1009109

**Published:** 2022-12-07

**Authors:** Yafei Shen, Jing Li, Fangfang Chen, Ruimei Cheng, Wenfa Xiao, Lichao Wu, Lixiong Zeng

**Affiliations:** ^1^ Ecology and Nature Conservation Institute, Chinese Academy of Forestry, Key Laboratory of Forest Ecology and Environment of National Forestry and Grassland Administration, Beijing, China; ^2^ Co-Innovation Center for Sustainable Forestry in Southern China, Nanjing Forestry University, Nanjing, China; ^3^ Key Laboratory of Soil and Water Conservation and Desertification Combating of Hunan Province, College of Forestry, Central South University of Forestry and Technology, Changsha, China

**Keywords:** subtropical, soil physical and chemical properties, forest stand types, coupling characteristics, survival competition

## Abstract

As a key component of terrestrial ecosystems, soil interacts directly with aboveground vegetation. Evaluating soil quality is therefore of great significance to comprehensively explore the interaction mechanism of this association. The purpose of this study was to fully understand the characteristics of aboveground vegetation, soil quality, and their potential coupling relationship among different forest types in Hunan Province, and to provide a theoretical basis for further exploring the mechanisms underlying soil–vegetation interactions in central China. We have set up sample plots of five kinds of forests (namely broad-leaved forest, coniferous forest, coniferous broad-leaved mixed forest, bamboo forest, and shrub forest) in Hunan Province. To explore the differences of vegetation characteristics and soil physical and chemical properties among the five stand types, variance analysis, principal component analysis, and regression analysis were used. Finally, we explored the coupling relationship between soil quality and aboveground vegetation characteristics of each forest. We found that there were significant differences in soil quality among the forest types, ranked as follows: shrub forest > bamboo forest > broad-leaved forest > mixed coniferous and broad-leaved forest > coniferous forest. In general, there was a negative correlation between vegetation richness and soil quality in the broad-leaved forest and the shrub forest, but they showed a positive correlation in the coniferous forest, the mixed coniferous and broad-leaved forest, and the bamboo forest. As a necessary habitat condition for aboveground vegetation, soil directly determines the survival and prosperity of plant species. These results indicated that for vegetation–soil dynamics in a strong competitive environment, as one aspect wanes the other waxes. However, in a weak competitive environment, the adverse relationship between vegetation and soil is less pronounced and their aspects can promote.

## 1 Introduction

As the largest carbon pool on Earth’s land surface, soil participates in many ecological processes underpinning forest succession and is a crucial habitat factor for the survival of aboveground vegetation (Crowther et al., 2019; Li et al., 2021). Soil has many physical and chemical properties, which can directly or indirectly affect vegetation, thereby leading to the establishment or enrichment of aboveground vegetation (Russell and Beauchamp, 2017). Research has shown that the presence of soil organic matter confers high porosity, which can improve soil water retention and soil fertility and promote the growth of trees (Norris et al., 2014). Soil temperature influences plant root biomass and drives how soil moisture content and pH value impact tree growth, which creates the conditions determining tree survival rates (Greenwood et al., 2015; Nishar et al., 2017). Liu et al. (2020) studied the relationship between *Pinus taiwanensis* Hayata seedling regeneration and soil nitrogen, finding that its survival rate was positively correlated with soil nitrogen, and tree growth was directly correlated with the availability of nitrogen elements (Liu et al, 2017). Maia et al. (2020) studied the sensitivity of aboveground vegetation abundance to climate and soil in seasonal arid tropical forests and pointed out that almost all vegetation attributes were significantly correlated with soil. Conversely, the characteristics of aboveground vegetation are also key factors capable of affecting soil physical and chemical properties, and the selection and complementarity between plants are beneficial to soil carbon storage and nutrient turnover (Loreau and Hector, 2001; Haghverdi and Kooch, 2019). Studies have shown that the dynamic cycling of soil carbon and nitrogen differs among different plant types, whereas higher plant diversity characteristics can augment soil carbon storage and nutrient turnover (Cong et al., 2014; Lange et al., 2015). Dawud et al. (2017) found that soil carbon storage in different soil layers was significantly and positively correlated with vegetation diversity. The research of Pardo et al. (2013) showed that divergent nutrient absorption strategies are the key to understanding changes to vegetation–soil nutrient cycle characteristics. Tumushime et al. (2019) found from the perspective of competition that the competition of trees in the forest would affect the accumulation of soil organic matter. Collectively, the studies cited above are helpful for better understanding the interactions between vegetation and soil physical/chemical properties. However, the diversity and complexity of forest ecosystems, the variety of their stand types, and an uneven stand status lead to differences in the physical and chemical properties of forest soil at regional or local scales (Jansson and Tas, 2014). Soil quality, being an inherent property of soil, is a comprehensive reflection of soil physical and chemical properties and is involved in pivotal processes in forest ecosystems, such as carbon storage and biomass production (Obade and Lal, 2016; Huang et al., 2018; Obade, 2019). In tandem, the characteristics of aboveground vegetation play an important role in improving soil quality (Zhang et al., 2019). Therefore, taking soil quality as a comprehensive index to evaluate soil physical and chemical properties, and exploring the correlation between soil quality and aboveground vegetation distribution characteristics of different forest types on this basis, may hold the key to elucidating the underlying ecological mechanism(s) of plant–soil interaction systems.

Concerning the evaluation system for soil quality, some scholars have shown a linear relationship between soil quality fraction and measured data that is sensitive to soil changes, so a linear scoring model may be used to evaluate soil quality (Liu et al., 2014). Yet, other researchers found no obvious linear relationship between the soil quality score and soil physical and chemical indexes, so they instead argued for a non-linear scoring model to evaluate soil quality (Bi et al., 2013; Li et al., 2017a). Given that soil quality varies with geographical location and spatial scale, it is very important to select suitable soil quality evaluation methods that have been verified and can be compared across sites (Guo et al., 2017). Currently, the fuzzy mathematical model is the main conventional method to evaluate soil quality, along with the gray relational model method and principal component analysis (PCA) combined with GIS (Gruijter et al., 2011; Mondino et al., 2015; Vasu et al., 2016; Feng et al., 2018). In fact, PCA has been the most widely used method in quantitative evaluations of soil quality.

Hunan Province in China has rich and diverse forest types, rapid vegetation recovery, and high reserve of forest resources, which play an extremely important role in water conservation and maintaining the regional carbon cycle and ecological balance in the Yangtze River Basin (Liu et al., 2016). In the existing studies in this area, there are few important findings about the correlation between soil quality and aboveground vegetation characteristics. However, the characteristics of aboveground vegetation and the competition relationship between them will affect soil carbon and nitrogen and other physical and chemical factors to a certain extent (Wang et al., 2020a). Will this become an important potential ecological factor in the soil vegetation interaction mechanism? Accordingly, five forest types—broad-leaved forest, coniferous forest, mixed coniferous and broad-leaved forest, bamboo forest, and shrub forest—were selected in Hunan Province and their respective soil sampled. PCA was used to reduce into a few dimensions the data on soil bulk density, porosity, pH value, organic matter, total nitrogen, total phosphorus, and total potassium to construct a total data set (TDS). Then, we calculated the respective weights of the physical and chemical properties of each forest type’s soil. To perform this calculation, the membership function was introduced to comprehensively evaluate the soil quality of the various forest types. Based on this analysis, the coupling relationship between soil quality and aboveground vegetation density and tree height characteristics was analyzed to explore the relative fitting degree of soil quality and vegetation characteristics of the five forest types in Hunan Province. The results can provide a theoretical reference for the soil nutrient management and forest management model in this region and provide a theoretical basis for further exploring the mechanism of soil–vegetation interactions in central China.

## 2 Materials and methods

### 2.1 Study region

Hunan Province is located in the middle of a subtropical zone within a single bioclimatic zone. It is one of the most important forest areas in south China, distinguished by superior natural conditions, a suitable climate, many economic trees, and rapid growth. Having a continental subtropical monsoon humid climate, there are four distinct seasons in Hunan Province: a changeable spring temperature, damp and rainy; a long summer hot period, warm and wet heavy; a dry autumn period; and a short winter cold period. The zonal soils here are mainly red soil and yellow soil, respectively, in the land east and west of the Xuefeng Mountains in Wulingyuan. The main trees are *Quercus glauca*, *Cunninghamia lanceolata*, and *Pinus massoniana*. The main shrubs are *Camellia oleifera*, *Lindera glauca*, and *Rhododendron simsii*. The main herbs are *Arthraxon hispidus*, *Ophiopogon japonicas*, and *Woodwardia japonica*. The landscape is dominated by hills, these mainly distributed in its eastern, southern, and western parts (Liu et al., 2016).

### 2.2 Methodology

#### 2.2.1 Plot selection and investigation

During the period of 2015–2016, according to the site conditions in Hunan Province and its present distribution of forest resource types, we selected main typical site conditions and ecological locations of important regional zonal vegetation types. In particular, the distribution of forestland with larger areas and having relatively few signs of natural- or human-disturbed soils was chosen; these sampling locations are shown in **Table 1**. In each forest type, 5–15 plots (10 m × 10 m) were established to identify the dominant tree species and measure each number of trees, tree DBH, and tree height (among them, tree basal area = 1/4π(DBH)^2^). The height and coverage of the main dominant shrubs were also investigated and recorded. At the same time, local environmental factors such as elevation, slope aspect, and canopy density were recorded. The sampling design and effort at the sites in the study area are detailed in **Table 2**.

**Table 1:** Site selection and quantity of plots in Hunan Province, China.

City	Longitude	Latitude	Number of plots
Changsha	112°36′~113°35′E	28°00′~28°36′N	9
Yizhang	112°88′~112°99′E	24°93′~24°99′N	15
Dongkou	110°19′~110°44′E	26°99′~27°15′N	8
Longshan	109°65′~109°66′E	29°58′~29°62′N	4
Pingjiang	113°88′~113°94′E	26°58′~28°60′N	7
Sangzhi	110°05′~110°08′E	29°69′~29°78′N	6
Shimen	110°77′~110°87′E	29°95′~30°11′N	4
Taojiang	112°17′~112°28′E	28°25′~28°54′N	5
Shufu	110°50′~110°57′E	27°77′~27°91′N	5
Xinhua	110°30′~111°31′E	28°14′~28°19′N	7
Chaling	113°47′~113°50′E	26°78′~27°29′N	6
Hengshan	112°69′~112°75′E	27°28′~28°28′N	5
Shuangpai	110°91′~110°96′E	26°08′~26°43′N	5
Xiangtan	112°77′~112°86′E	27°50′~27°93′N	5

**Table 2:** Basic information of plots of different forest land types.

Types of woodland land	Origin	Number of plots	Succession stage	Altitude (m)	Large terrain	Small terrain	Canopy	Coverage
I	Plantation Natural forests	5 20	Forest stage	45~1533	Mountains; hills	Slopes; flats	0.3~0.95	6%~90%
II	Plantation Natural forests	21 2	Forest stage	85~1102	Mountains; hills	Slopes	0.2~0.95	4%~80%
III	Plantation Natural forests	17 14	Forest stage	60~1623	Mountains; hills	Slopes	0.1~0.95	2%~90%
IV	Plantation Natural forests	4 13	Forest stage	50~1795	Mountains; hills	Slopes; flats	0.6~0.98	1%~50%
V	Plantation Natural forests	0 6	Shrub stage	480~1835	Mountains	Slopes	0	2%~90%

I: broad-leaved forest; II: coniferous forest; III: mixed coniferous and broad-leaved forest; IV: bamboo forest; V: shrub forest. The same below.

#### 2.2.2 Soil sampling and determination of physical and chemical factors

In each 10 m × 10 m quadrat, following forest soil survey technical regulations (Technical regulations for forest soil survey, 2014), five soil profiles were set at the four vertices and the intersection of the diagonals (i.e., the center point), using the five-point sampling method, to investigate the soil properties and texture. Stratified sampling was carried out as follows: 0–10, 10–20, and 20–40 cm, with soil samples collected from the same soil layer in various sides mixed together after removing branches, leaves, and litter. After air drying and grinding, soil samples were bagged after passing them through 2-, 1-, and 0.25-mm sieves and then stored in the laboratory until later use. Referring to the *Soil Agrochemical Analysis* (3^rd^ ed.) (Bao, 2000), soil pH value (potentiometric method), soil organic matter (potassium dichromate volumetric method), total nitrogen (Kjeldahl method), total phosphorus (sulfuric acid–perchloric acid elimination method), and total potassium (flame photometry) were each determined. In addition, undisturbed soil samples were collected with a 100-cm^3^ ring knife at the corresponding depth of each sampled soil profile in the sites (forest types) to measure and analyze their soil bulk density and soil porosity.

#### 2.2.3 Soil quality assessment

Due to their wide variety, the determination of soil quality indicators is complicated and varies considerably among different soil systems (Lal, 2016). Therefore, this study considered all the physical and chemical properties of the soil measured, and the soil quality index (SQI) was established by the total data set (TDS) method. Soil quality evaluation was completed in four steps.

First, the original data of soil physical and chemical indexes were weighted to obtain the average value of soil property indexes in a given sampling site. Soil bulk density (SD), soil porosity (SP), potential of hydrogen (pH), soil organic matter (SOM), soil total nitrogen (STN), soil total phosphorus (STP), and soil total potassium (STK) were normalized to make the evaluation indexes comparable (Feng et al., 2018). Membership function was used to score each, whose corresponding membership value was calculated according to the method of Masto et al. (2008) (i.e., a soil quality linear scoring model). For the soil pH value, its neutral value (7.0) is considered desirable. In our soil survey, because all values were<7, this index is considered as “higher is better”. To sum up, soil porosity, pH value, organic matter, total nitrogen, total phosphorus, and total potassium belonged to the “higher, the better” type equation, whereas soil bulk density belonged to the “lower, the better” type equation, whose specific calculation formulas are as follows:


(1) $$Q_{1}=\frac{X-X_{min}}{X_{max}-X_{min}}$$

(2) $$Q_{2}=\frac{X_{max}-X}{X_{max}-X_{min}}$$

where *Q* represents the degree of membership (0–1), *X* represents the measured value of the soil index, *X_min_* represents the minimum value of the soil index, and *X_max_* represents the maximum value of the soil index. Equation (1) is an index scoring function of “the higher, the better” (*Q_1_*), and Equation (2) is an index scoring function of “the lower, the better” (*Q_2_*).

Next, the common factor variance obtained *via* PCA using all the measured soil property data was used to express the contribution of an index to the overall variance: the larger it is, the greater its contribution to the overall variance (Chen et al., 2019). We used PCA to calculate the weight value of each TDS indicator, where weight is equal to the ratio of the common factor variance of each indicator to the sum of the common factor variance of all indicators (Rahmanipour et al., 2014). The calculation for that is shown in Formula (3). Among them, *c_i_* represents the common factor variance for each soil factor, and *W_i_* represents the index weight value.


(3) $$W_{i}=\frac{c_{i}}{\sum_{i=1}^{n}c_{i}}$$

(4) $$\text{SQI}=\sum_{i=1}^{n}W_{i}Q_{i}$$

Finally, the soil quality index (SQI) under each forest according to Formula (4) is calculated, where *Q_i_* represents the degree of membership, *n* is the number of indicators, and *W_i_* represents the index weight value, and the higher the SQI value, the better the soil quality (Doran and Parkin, 1994).

### 2.3 Data

#### 2.3.1 Analysis of variance significance

The statistical analysis of the aboveground vegetation data was performed by one-way ANOVA to represent the aboveground vegetation distribution characteristics of different forest land types. The statistical analysis of the data of the same forest land but different soil layers was performed by one-way ANOVA to reflect the differences in soil characteristics between different soil layers of the same forest stand. Similarly, the same analysis was performed on the data of the same soil layer but different forest land to reflect the differences of soil characteristics between different forest types in the same soil layer. Based on this, the SQI mean of three soil layers in each forest was calculated, and one-way ANOVA was performed to test for the soil quality differences among different forests. Duncan’s multiple-range test was performed to detect the statistical significance between treatments. *P* < 0.05 was considered statistically significant.

#### 2.3.2 Principal component analysis

We conducted principal component analysis on each soil physical and chemical factor, calculated its corresponding common factor variance, and calculated the weight of these factors according to Formula (3). Finally, the SQI of each sample was obtained according to Formula (4). PCA was used as described in Subsection 2.2.3.

#### 2.3.3 Regressive analysis

In order to exclude collinearity among aboveground vegetation features, we first used Pearson correlation analysis to obtain their correlation features. Considering the ecological role of these indicators comprehensively, we chose to delete some indicators, and the remaining indicators would be used as independent variables in regression analysis with the SQI. General linear regression analysis was used to explore the potential linear relationship between the SQI of each forest land type and the distribution characteristics of aboveground vegetation, so as to reflect the impact of comprehensive soil quality on the survival of aboveground vegetation. Data analysis and plotting were implemented by R 4.0.3 software (R core team, 2019).

## 3 Results

### 3.1 Distribution characteristics of aboveground vegetation in different forest types

**Figure 1:**
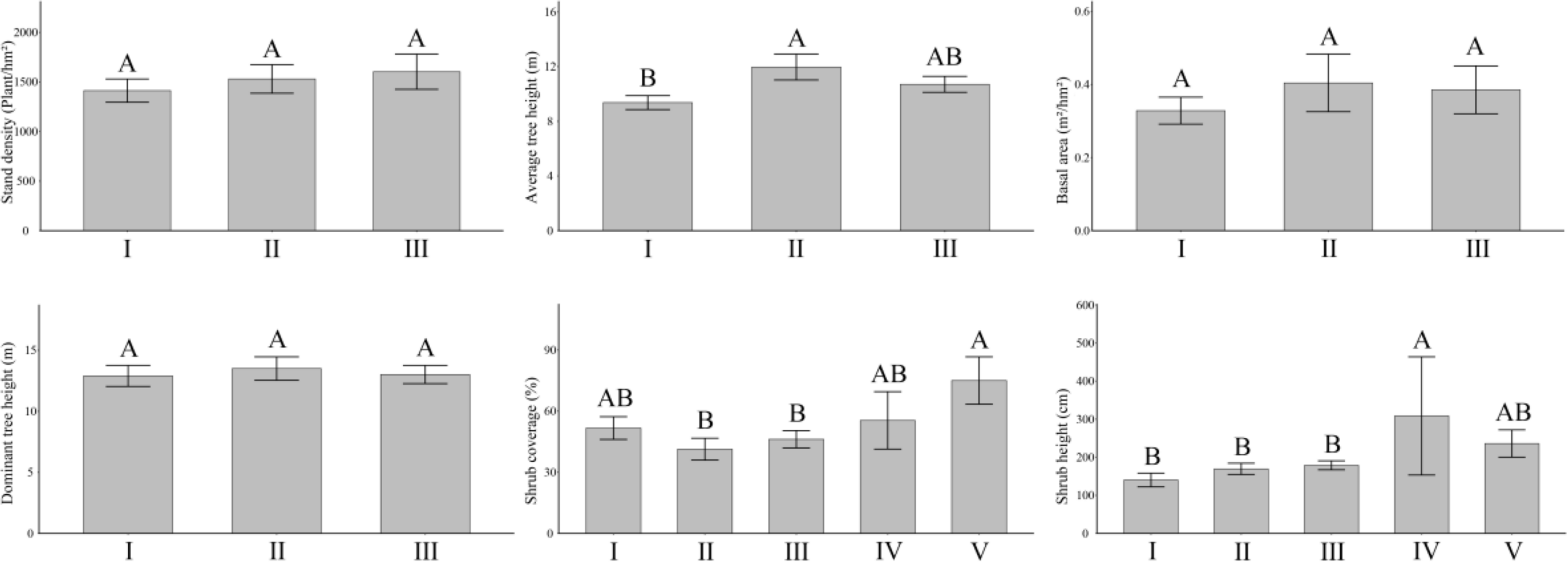
Distribution difference of aboveground vegetation in different forest land types. Above the error line, different capital letters indicate that there are significant differences among different forest land types. I, broad-leaved forest; II, coniferous forest; III, mixed coniferous and broad-leaved forest; IV, bamboo forest; V, shrub forest. The same below.

Among the forest types in Hunan Province, the aboveground vegetation index characteristics differed (**Figure 1**). In general, the average tree height was 9.37 to 10.70 m and ranked as coniferous forest > mixed coniferous and broad-leaved forest. Shrub coverage was ranked as follows: shrub forest > bamboo forest > broad-leaved forest > mixed coniferous and broad-leaved forest > coniferous forest (41.30%–75.00%). The ranking for shrub height was bamboo forest > shrub forest > mixed coniferous and broad-leaved forest > coniferous forest > broad-leaved forest, ranging from 140.21 to 308.57 cm. In addition, the stand density was ranked from 1,412.5 to 1,603.33 plants/hm^2^, the basal area was ranked from 0.33 to 0.40 m^2^/hm^2^, and the dominant tree height was ranked from 12.90 to 13.50 m, but there was no significant difference among each forest type.

According to **Table S1**, the average tree height of the coniferous forest (11.97 m) was significantly (*P*< 0.05, the same below) higher than that of the broad-leaved forest (9.37 m). The shrub coverage was significantly higher in the shrub forest (75.00%) than the coniferous forest (41.30%) or the mixed coniferous forest (46.17%). The shrub height of the bamboo forest (308.57 cm) was significantly higher than that of the broad-leaved forest (140.21 cm), the coniferous forest (169.52 cm), and the coniferous and broad-leaved mixed forest (178.87 cm). There were no significant differences in stand density, basal area, and dominant tree height among broad-leaved, coniferous, and mixed coniferous forest types (*P* > 0.05, the same below).

That the vegetation indices differed among forest types indicated that vegetation growth is locally adapted to the environment. Forest type is the key factor leading to the distribution and growth characteristics of aboveground vegetation.

### 3.2 Characteristics of soil physicochemical properties in different forest types

**Figure 2:**
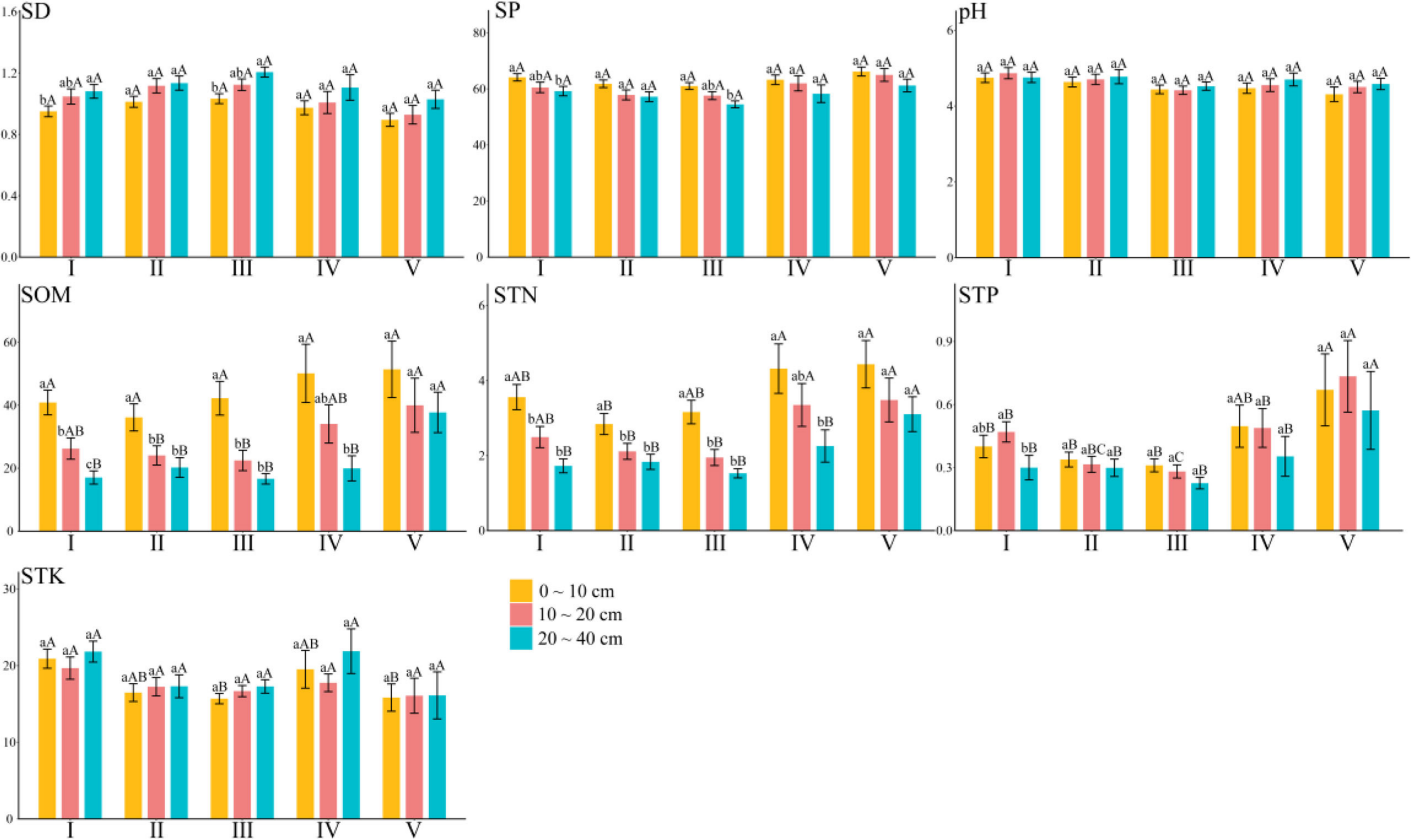
Differences of soil physical and chemical properties in different soil layers of different forest land types. SD, soil bulk density; SP, soil porosity; pH, pH value; SOM, soil organic matter; STN, soil total nitrogen; STP, soil total phosphorus; STK, soil total potassium. Above the error line, different lowercase letters indicate significant differences between different soil layers; different capital letters indicate that there are significant differences among different forest land types. I, broad-leaved forest; II; coniferous forest; III, mixed coniferous and broad-leaved forest; IV, bamboo forest; V, shrub forest. The same below.

**Figure 2** shows the distribution differences of soil physical and chemical properties in different soil layers of the forest types in Hunan Province. Overall, there were differences in seven soil physical and chemical indexes among the forest types. The mean value of soil bulk density, porosity, pH, organic matter, total nitrogen, and total phosphorus varied from 1.02 to 1.11 g·cm^-3^, 58.10% to 64.47%, 4.45 to 4.78, 27.37 to 43.92 g·kg^-1^, 2.30 to 3.76 g·kg^-1^, and 0.28 to 0.67 g·kg^-1^, respectively. For total potassium, its mean ranged from 15.99 to 20.85 g·kg^-1^.

In the broad-leaved forest and also the mixed coniferous and broad-leaved forest, soil bulk density was significantly higher in the 20–40 cm than 0–10 cm soil layer, whereas the soil porosity was significantly lower in the 20–40 cm than 0–10 cm soil layer.

For soil organic matter, it was significantly different among the three layers of the broad-leaved forest. In the coniferous forest and the coniferous–broad-leaved mixed forest, the soil organic matter content of the 0–10 cm soil layer is significantly higher than that of the other two. The 0–10 cm soil layer of the bamboo forest was significantly higher than that of the 20–40 cm soil layer, whereas the soil layers in the shrub forest were similar to each other.

The total nitrogen in the 0–10 cm soil layer of the broad-leaved forest, the coniferous forest, and the mixed coniferous and broad-leaved forest was significantly higher than that in the other two soil layers, and that in the 20–40 cm soil layer of the bamboo forest was significantly lower than that in the other two soil layers, whereas it did not differ significantly among the soil layers in the shrub forest.

The total phosphorus in the 10–20 cm layer of the broad-leaved forest was significantly higher than that in the 20–40 cm layer, but there was no significant difference among the soil layers in the coniferous forest, the mixed coniferous and broad-leaved forest, the bamboo forest, and the shrub forest.

For total potassium, there were no significant differences among soil layers in the forest types. Total phosphorus increased first and then decreased in the broad-leaved forest and the shrub forest, and total potassium decreased first and then increased in the broad-leaved forest and the bamboo forest. However, as the soil layer deepened, the content of soil organic matter and total nitrogen decreased. This indicated that the non-metallic nutrient elements accumulate more in the surface soil, whereas metallic nutrient elements ended up underground, but the overall range in their variation is small.

At the regional scale, forest type had no significant effect upon soil bulk density, porosity, or the pH of each soil layer. The soil organic matter content of the 10–20 cm soil layer was significantly higher in the shrub forest than either the coniferous forest or the mixed coniferous and broad-leaved forest, and that of the 20–40 cm soil layer was significantly the greatest in the shrub forest, following this ranking: shrub forest > bamboo forest > mixed coniferous and broad-leaved forest > broad-leaved forest > coniferous forest.

The total nitrogen content of the 0–10 cm soil layer was significantly lower in the coniferous forest than the bamboo forest or the shrub forest, and that of the 10–20 cm soil layer in the shrub forest and the bamboo forest significantly exceeded that of the coniferous forest and the mixed coniferous and broad-leaved forest, whereas that of the 20–40 cm soil layer in the shrub forest was significantly higher than all other forest types. The relative content of total nitrogen was ranked as follows: shrub forest > bamboo forest > broad-leaved forest > mixed coniferous and broad-leaved forest > coniferous forest.

The total phosphorus in the 0–10 cm soil layer is significantly higher in the shrub forest than in the broad-leaved forest, the coniferous forest, and the mixed coniferous and broad-leaved forest. The total phosphorus in the 10–20 and 20–40 cm soil layers of the shrub forest is significantly higher than the other four forest types, with the relative content of sorting given as follows: shrub forest > bamboo forest > broad-leaved forest > coniferous forest > mixed coniferous and broad-leaved forest.

The soil total potassium content of the 0–10 cm soil layer in the mixed coniferous and broad-leaved forest was significantly lower than that in the broad-leaved forest, the bamboo forest, and the shrub forest, but there was no significant difference between the 10–20 and 20–40 cm soil layers. These results indicated that forest type is the main factor leading to the distribution of soil physical and chemical properties.

### 3.3 Evaluation of soil quality in different forest types

**Table 3** presents the results for the PCA used to comprehensively evaluate the soil quality of the forest types. According to the principle of eigenvalue >1, the first two principal components were selected, whose eigenvalue was 3.846 and 1.226, respectively. The variance explained by the first principal component was 54.937%, whereas that of the second principal component was 17.508%, totaling 72.445%. This indicated that the two extracted principal components reflected well the comprehensive information from the evaluation indexes. Soil bulk density, porosity, organic matter, total nitrogen, and total phosphorus played key roles in the first principal component, whereas pH and total potassium largely underpinned the second principal component. According to **Table 3**, the weights of the seven soil bulk density indexes in descending order were 0.162, 0.163, 0.151, 0.160, 0.174, 0.137, and 0.053.

**Table 3:** Results of principal component analysis, factor loadings, common factor variance, and indicators’ weight.

Soil properties	PC1	PC2	Common factor variance	Weights
SD	-0.904	0.077	0.824	0.162
SP	0.905	-0.077	0.825	0.163
pH	-0.229	0.844	0.765	0.151
SOM	0.900	-0.030	0.810	0.160
STN	0.935	0.087	0.882	0.174
STP	0.680	0.484	0.697	0.137
STK	-0.100	0.509	0.269	0.053
Eigen values	3.846	1.226		
Variance contribution	54.937%	17.508%		
Cumulative variance contribution	54.937%	72.445%		

SD, soil bulk density; SP, soil porosity; pH, pH value; SOM, soil organic matter; STN, soil total nitrogen; STP, soil total phosphorus; STK, soil total potassium.

**Figure 3:**
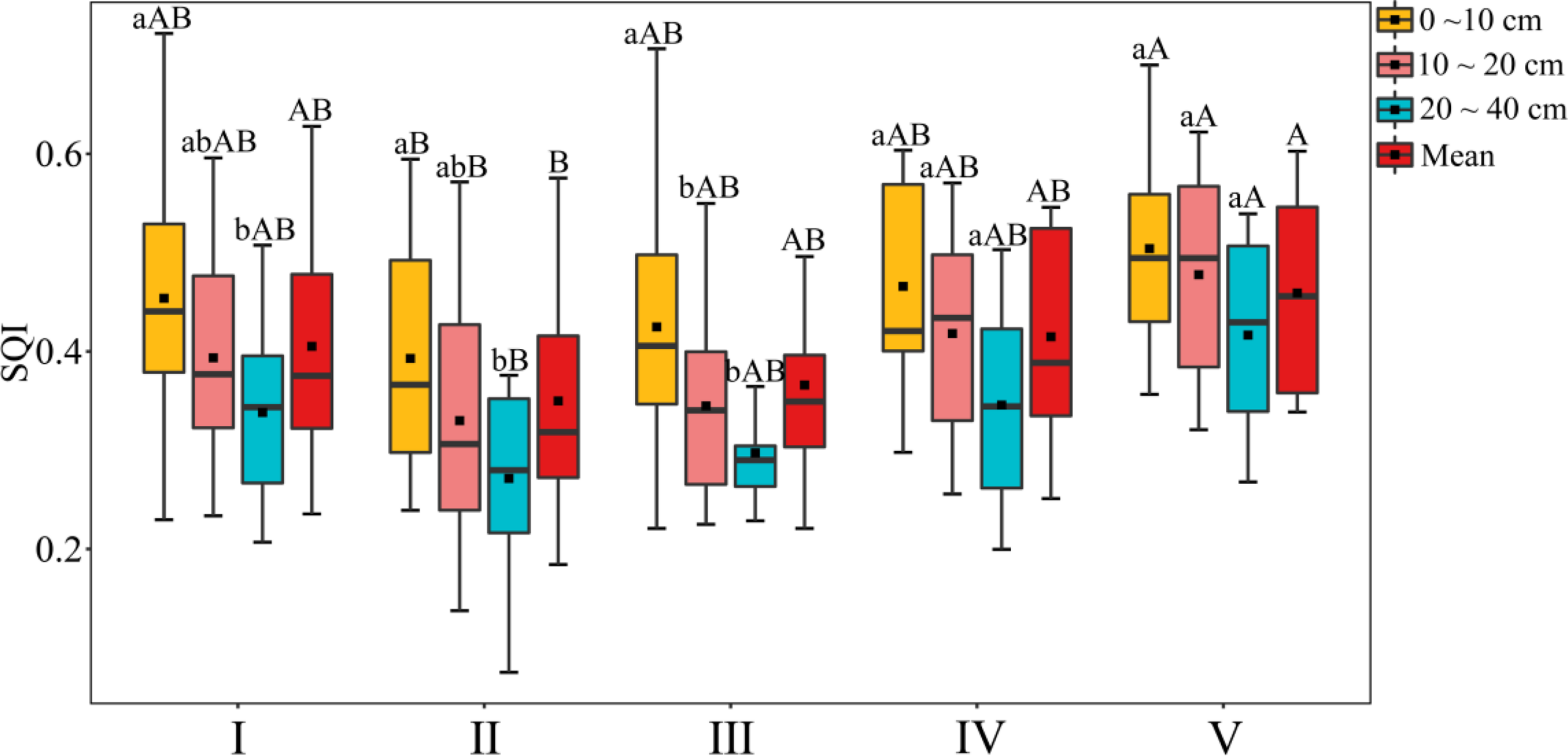
Comprehensive evaluation results of soil quality of different forest land types. Above the error line, different lowercase letters indicate significant differences between different soil layers, and different capital letters indicate that there are significant differences among different forest land types. I, Broad-leaved forest; II, coniferous forest; III, mixed coniferous and broad-leaved forest; IV, bamboo forest; V, shrub forest. The same below.

According to the weight of each soil physical or chemical property, a soil comprehensive quality index (SQI) for each soil layer in each forest type can be calculated by substituting it into Formula (4). The results are shown in **Figure 3**. In the broad-leaved forest and the mixed coniferous and broad-leaved forest, the SQI differed significantly differently among the three soil layers and decreased with increasing soil depth in all forest types. There were significant differences in the mean value of the SQI among the forest types, being significantly higher in the shrub forest than the coniferous forest. The SQI, ranked from high to low, was as follows: shrub forest > bamboo forest > broad-leaved forest > mixed coniferous and broad-leaved forest > coniferous forest.

### 3.4 Correlations between soil quality and aboveground vegetation characteristics

**Figure 4:**
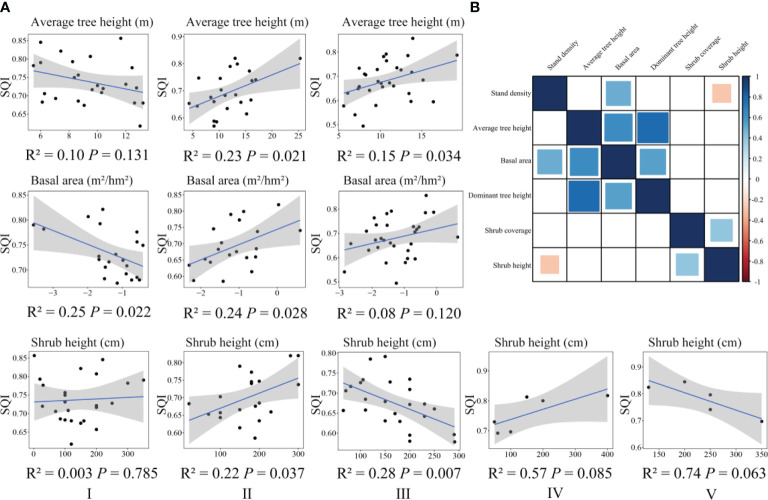
Correlation between soil quality and vegetation characteristics in different forest land types **(A)**. Correlation of aboveground vegetation characteristics of different forest types **(B)**. The blue square indicates that there is a significant positive correlation between indicators; the red square indicates that there is a significant negative correlation between indicators; the white square indicates that there is no significant correlation between indicators. I, broad-leaved forest; II, coniferous forest; III, mixed coniferous and broad-leaved forest; IV, bamboo forest; V, shrub forest.

We determined the aboveground vegetation index ultimately used for general linear regression analysis (**Figure 4B**) and conducted general linear regression analysis with SQI (**Figure 4A**). In the broad-leaved forest, the SQI was significantly negatively correlated with the basal area. The SQI of the coniferous forest was significantly positively correlated with average tree height, basal area, and shrub height. In the mixed coniferous and broad-leaved forest, the SQI was significantly positively correlated with average tree height and significantly negatively correlated with shrub height. In the bamboo forest and the shrub forest, the SQI was not significantly correlated with shrub height.

The fitted linear relationships between the SQI and aboveground vegetation characteristics in the different forest types reflected the changes in the trends of the two indexes in the increase and decrease and reflected the potential ecological correlation characteristics of vegetation–soil quality interactions.

## 4 Discussion

### 4.1 Evaluation of soil quality in the forest types

As an important structural component of forest ecosystems, the physical and chemical properties of soil are the comprehensive embodiment of forest soil nutrients, and they play a pivotal role in ensuring vegetation growth and preventing soil erosion (Davis et al., 2018; Huang et al., 2019). Among them, soil bulk density is regarded as the key factor governing soil physical and chemical properties as well as soil compactness (Al-Shammary et al., 2018). By contrast, soil porosity mainly mediates the transport of soil water, the exchange of soil gases, and the movement and storage of soil nutrients. It also affects soil microbial activity and promotes the absorption and growth abilities of plant roots, both being important components of soil physical and chemical properties (Luna et al., 2018). In this study, in general, the soil bulk density of each forest land type had an increasing trend with the deepening of the soil layer, but the porosity results were opposite. This may be related to humus accumulation and the distribution of plant roots and their interactions with the surrounding soil environment: in particular, shallow soil accumulated more humus and developed a denser root system, so its soil environmental improvement is more pronounced (Reich et al., 2003; Shao et al., 2020). Soil pH participates in the regulation of soil biogeochemical processes and exerts a cascading effect on the stabilization of soil ecosystem structure and function (Hong et al., 2018). In this study, the pH values of all forest types’ soil were<7, implying weak acidity overall, but there was no significant difference in pH among all soil layers in any of the forest types. This may be due to the short succession time of the selected forest region, being still a “young forest”, leaving few if any significant differences detectable in its physical and chemical properties of soil (Li et al., 2017b).

Among soil nutrient indexes, soil organic matter, as the basis of soil physical and chemical properties, promotes the formation of soil aggregates and maintains the nutrient cycling of the forest ecosystem (Feng et al., 2016; Yuan et al., 2018). At the same time, total nitrogen, total phosphorus, and total potassium in soil can reflect the total nutrient storage level in the soil nutrient pool (Rboertson and Vitowsek, 1981). We found that there were differences among forest land types and soil layers, and some reached a significant level. The differences in soil organic matter, total nitrogen, and total phosphorus had the pattern shrub forest > bamboo forest > tree forest, perhaps because the shrub forest protected soil organic carbon and delayed its decomposition rate, thus conferring to it a strong carbon fixation ability (An et al., 2010; Yang et al., 2020). The pronounced soil organic matter content in the bamboo forest may be caused by this forest type’s high input of organic matter as litter (Fukushima et al., 2015). It is known that the accumulation of organic matter is closely related to the soil nutrient supply capacity of nitrogen and phosphorus, which is also a likely reason for the overall consistent trend of the above nutrient contents of the forest types in this study (Dong et al., 2021). The content of total potassium in the broad-leaved forest and the bamboo forest was relatively high, likely due to higher litter inputs in the broad-leaved forest and the bamboo forest compared with other forest types (Qiao et al., 2014). Due to the influence of soil parent material, soil organic matter, and total nitrogen decrease with the increase in soil depth, our findings are similar to those of Wang et al. (2020b).

The SQI is a composite index rather than a value derived from a single indicator (Askari and Holden, 2015). The above soil physical and chemical properties can be used to build a regional soil quality index (SQI) through the calculation of membership degree and weight. Weight calculation results showed that total nitrogen was assigned the greatest weight, reaching a proportion of 0.174, probably because of nitrogen’s critical status in the soil nutrient cycle. Studies have shown that nitrogen is not only an important soil nutrient element but also the key factor limiting plant growth, and its effectiveness and absorption can differ according to the external environment and has changed dramatically (Ågren et al., 2012; Kuster et al., 2016). Furthermore, the weights assigned to soil organic matter, soil bulk density, and porosity also accounted for a considerable proportion; hence, there are also key factors contributing to the SQI’s determination (Mariscal et al., 2007). In this study, the soil quality of the shrub forest was significantly higher than that of other forest types, followed by the bamboo forest. In the forest types with trees, the SQI generally increased with the succession stage of the area. We found that the soil quality in the shrub forest is relatively high, which is due to the fact that shrubs within a certain coverage range can concentrate nutrients around themselves by changing factors such as soil structure and humidity, forming a “Fertility islands effect.” In other words, the soil in the shrub forest with appropriate coverage has higher soil nutrient content, thus improving the soil quality (Luca et al., 2008). The soil quality of the bamboo forest was relatively high, which might have been augmented by human disturbance and management measures, and the trends in the changes to soil quality of the other three types of arbor forest are consistent with the positive succession of the same region (Feng et al., 2018). Soil quality was greater in the broad-leaved forest than the other two arbor forest types because the broad-leaved forest in this region was in the later successional stage, once featuring richer species composition, a more complex community structure, and more efficient nutrient enrichment and cycling (Feng et al., 2016). The soil quality decreased through the soil layers, which was consistent with the trends for changes to soil total nitrogen, organic matter, bulk density, and porosity among different soil layers. Therefore, the comprehensive comparison and evaluation of our soil quality results are basically consistent with the findings of Wang et al. (2020b) in central China.

### 4.2 Coupling relationship between soil quality and aboveground vegetation characteristics

The Niche theory predicts that plant species’ growth rates are adapted to a specific environment, thus presenting different habitat preferences, and the survival and growth of trees are also an important embodiment of habitat conditions (Potts et al., 2002; Itoh et al., 2003; Zhang et al., 2017). As a carrier, soil participates in the process of material and energy exchange and circulation in terrestrial ecosystems, providing necessary water and nutrients for the growth of aboveground vegetation, and creates favorable habitat conditions, thus affecting forest regeneration and succession (Holmes, 2010). In tandem, the structure and diversity of the vegetation community can negatively influence soil formation, soil structure, and soil nutrient status (Cerqueira et al., 2012; Bloor and Bardgett, 2012). Here, we analyzed the coupling relationship between soil quality and aboveground vegetation characteristics—including average tree height, basal area, and shrub height—of the five forest types in Hunan Province. We found that, in the broad-leaved forests, soil quality was significantly negatively correlated with the basal area. This is because the broad-leaved forest in the subtropical region is in the late stage of succession. Due to density constraints, there is a strong competition between adjacent individuals of tree and shrub species, which excessively consumes nutrients in the soil (Dijkstra et al., 2010). At the same time, such a result affects soil microbial metabolism, changes soil structure and nutrient cycle efficiency, and further reduces soil quality (Kuzyakov, 2002; Zhu et al., 2010).

The positive effect of shrub height on soil quality may be the result of the competition after natural thinning and the improvement of soil quality by the winner (Luca et al., 2008). The consumption of soil quality is used by competition, and the result of its reduction is used to ensure the survival of the dominant tree species in this forest type. In the coniferous forest and the coniferous broad-leaved mixed forest, the soil quality was positively correlated with the average tree height and the basal area and has reached a significant level in general. This is due to the fact that the two forest land types are in the middle of succession, and the competition between trees is not intense; thus, the soil quality shows synchronous changes with the basal area and the average tree height. Interestingly, we found that there was a significant positive correlation between shrub height and soil quality in the coniferous forests, but the opposite was reflected in the coniferous broad-leaved mixed forests. This is because the mixed forest enriches the diversity of undergrowth vegetation and may overlap the niches of shrubs and some species, increase their competitive pressure, and then lead to declining soil quality, which cannot provide sufficient supply for their growth (Ammer, 2019). In the bamboo forest, shrub height has a positive effect on soil quality because the bamboo forest itself increased the input of organic matter to soil and strengthened the soil quality (Fukushima et al., 2015). In addition, the bamboo forest understory is relatively open, which provides a good light environment for vegetation growth there, mitigating competition pressure between species; hence, the shrub indexes and soil quality shifted in tandem (Song et al., 2017). In the shrub forest, shrub height has a negative effect on soil quality, likely due to the fierce competition in this forest caused by the huge amount of shrub vegetation present. Consequently, improved soil quality facilitated by the shrub’s presence alone is insufficient to compensate for their consumption of soil resources. In addition, as shrub coverage peaks with increasing density, water may become a limiting factor and its height growth could become inhibited, or self-thinning may occur due to the persistent and intense restriction between vegetation individuals (Ji et al., 2019).

In conclusion, the coupling between soil quality and aboveground vegetation in different forest types manifested distinct trends according to the resource competition dynamics across successional stages and the consumption of soil quality will give priority to the growth of dominant trees. Although this study used the soil physical and chemical properties of the whole data set, we did not fully consider various kinds of woodland soil nutrients and their innate ambient differences. Also ignored are trace elements necessary for plant growth and development and other chemical and biological factors, such as soil microbial community composition/activity, so our soil quality evaluation of the study area harbors certain inherent limitations. Finally, although we set up plots in different forest types, the age and structure of each differ to some extent, so their soil–vegetation interaction relationships warrant further study with both aspects incorporated in the sampling design.

## 5 Conclusion

The vegetation distribution of the five distinctive forest types in China’s subtropical Hunan Province arose from their habitat preferences in this climate zone. Soil, as a necessary habitat condition for aboveground vegetation, directly determines the persistence and fitness of plant species. Soil quality was evaluated based on the weighted calculation of soil physical and chemical properties. There were significant differences in soil physical and chemical properties among forest types and different soil layers. Soil total nitrogen, as a crucial nutrient in terrestrial ecosystems, accounted for a greater absolute proportion in the soil quality evaluation. Significant differences in soil quality were discernible among the forest types as follows: shrub forest > bamboo forest > broad-leaved forest > mixed coniferous and broad-leaved forest > coniferous forest; this ranking pattern is likely caused by changes to the soil physical and chemical properties of vegetation in differing successional stages associated with each forest type. In general, there was a negative correlation between vegetation richness and soil quality in the broad-leaved forest and the shrub forest. However, in the coniferous forest, the mixed coniferous and broad-leaved forest, and the bamboo forest, the competitive advantage of shrubs was still suppressed by strong dominant species, and there was positive correlation between vegetation and soil quality. As a necessary habitat condition for aboveground vegetation, soil directly determines the survival and prosperity of species. The nutrient return of litter can also supplement soil quality, thus enhancing the chances of vegetation survival. Under a strong competitive environment, vegetation and soil interact, whereby as one aspect wanes, the other waxes. By contrast, under a weak competitive environment, there is a milder relationship between vegetation and soil such that some of their aspects promoted each other. Looking ahead, our focus on soil quality evaluation data will shift to trace elements in soil, biological factors’ expansion, and the unification of forest age and structural dynamics, which is the key to further explore the soil–vegetation interactions in this region.

## Data availability statement

The original contributions presented in the study are included in the article/**Supplementary Material**. Further inquiries can be directed to the corresponding author.

## Author contributions

YS and JL drafted the manuscript. FC and LW participated in collecting the experiment data. RC and WX was involved in planning of study and designing of the work. The remaining authors contributed to refining the ideas, carrying out additional analyses and finalizing this paper. All authors contributed to the article and approved the submitted version.
