# Supplementary material for: Correlations between forest soil quality and aboveground vegetation characteristics in Hunan Province, China
Source: Front Plant Sci. 2022 Dec 7;13:1009109. doi: 10.3389/fpls.2022.1009109 (PMC9768340; doi:10.3389/fpls.2022.1009109)
Supplement: Supplementary file 1 [file Table_1.pdf]

Supplementary Material

Correlations between forest soil quality and aboveground vegetation characteristics in Hunan Province, China

Yafei Shen[§ a,b], Jing Li[§ a], Fangfang Chen[§ c], Ruimei Cheng [a,b] *, Wenfa Xiao [a,b], Lichao Wu [c], Lixiong Zeng [a,b]

Affiliations

a Ecology and Nature Conservation Institute, Chinese Academy of Forestry; Key Laboratory of Forest Ecology and Environment of National Forestry and Grassland Administration, Beijing 100091, China;
b Co-Innovation Center for Sustainable Forestry in Southern China, Nanjing Forestry University, Nanjing 210037, China;
c Key Laboratory of Soil and Water Conservation and Desertification Combating of Hunan Province, College of Forestry, Central South University of Forestry and Technology, Hunan, Changsha 410004, China.
E-mail addresses: yafeishen126@126.com (Y. Shen), ljingxuanxuan@163.com (J. Li), wulichao@sina.com (L. Wu), cafcheng@sina.com (R. Cheng), xiaowenf@caf.ac.cn (W. Xiao), zlxcaf@163.com (L. Zeng)

[§]Yafei Shen, Jing Li and Fangfang Chen contributed equally to this work.

*** Corresponding author**

Ruimei Cheng
E-mail: cafcheng@sina.com
Affiliation: Institute of Forest Ecology, Environment and Nature Conservation, Chinese Academy of Forestry, No. 2 of Dongxiaofu, Xiangshan Road, Haidian District, Beijing, 100091, China.
Tel.: +86 010 62889533

Table S 1 | Difference characteristics of aboveground vegetation in different forest types.

Forest land type	Stand density (Plant/hm^2)	Average tree height (m)	Basal area (m^2/hm^2)	Dominant tree height (m)	Shrub coverage (%)	Shrub height (cm)
I	1412.5±119.91a	9.37±0.52b	0.33±0.04a	12.90±0.88a	51.67±5.69ab	140.21±18.31b
II	1530.44±147.49a	11.97±0.97a	0.40±0.08a	13.50±0.98a	41.30±5.49b	169.52±15.15b
III	1603.33±180.73a	10.70±0.59ab	0.38±0.07a	13.01±0.76a	46.17±4.34b	178.87±12.01b
IV	-	-	-	-	55.43±15.23ab	308.57±167.67a
V	-	-	-	-	75.00±12.99a	236.00±40.25ab

Note: Different lowercase letters indicate significant differences between different soil layers. I: Broad-leaved Forest; II: Coniferous Forest; III: Mixed coniferous and broad-leaved Forest; IV: Bamboo Forest; V: Shrub Forest.
